# *S*. *mansoni Sm*KI-1 Kunitz-domain: Leucine point mutation at P1 site generates enhanced neutrophil elastase inhibitory activity

**DOI:** 10.1371/journal.pntd.0009007

**Published:** 2021-01-19

**Authors:** Fábio Mambelli, Bruno P. O. Santos, Suellen B. Morais, Enrico G. T. Gimenez, Duana C. dos S. Astoni, Amanda D. Braga, Rafaela S. Ferreira, Flávio A. Amaral, Mariana T. Q. de Magalhães, Sergio C. Oliveira

**Affiliations:** 1 Departamento de Genética, Ecologia e Evolução, Programa de Pós-Graduação em Genética, Instituto de Ciências Biológicas, Universidade Federal de Minas Gerais, Belo Horizonte, Minas Gerais, Brazil; 2 Laboratório de Imunologia de Doenças Infecciosas, Departamento de Bioquímica e Imunologia, Instituto de Ciências Biológicas, Universidade Federal de Minas Gerais, Belo Horizonte, Minas Gerais, Brazil; 3 Laboratório de Biofísica de Macromoléculas, Departamento de Bioquímica e Imunologia, Instituto de Ciências Biológicas, Universidade Federal de Minas Gerais, Belo Horizonte, Minas Gerais, Brazil; 4 Laboratório de Imunofarmacologia, Departamento de Bioquímica e Imunologia, Instituto de Ciências Biológicas, Universidade Federal de Minas Gerais, Belo Horizonte, Minas Gerais, Brazil; 5 Laboratório de Modelagem Molecular e Planejamento de Fármacos, Departamento de Bioquímica e Imunologia, Instituto de Ciências Biológicas, Universidade Federal de Minas Gerais, Brazil; 6 Instituto Nacional de Ciência e Tecnologia em Doenças Tropicais (INCT-DT), CNPq MCT, Salvador, Bahia, Brazil; Emory University, UNITED STATES

## Abstract

The *Schistosoma mansoni Sm*KI-1 protein is composed of two domains: a Kunitz-type serine protease inhibitor motif (KD) and a C-terminus domain with no similarity outside the genera. Our previous work has demonstrated that KD plays an essential role in neutrophil elastase (NE) binding blockage, in neutrophil influx and as a potential anti-inflammatory molecule. In order to enhance NE blocking capacity, we analyzed the KD sequence from a structure-function point of view and designed specific point mutations in order to enhance NE affinity. We substituted the P1 site residue at the reactive site for a leucine (termed RL-KD), given its central role for KD’s inhibition to NE. We have also substituted a glutamic acid that strongly interacts with the P1 residue for an alanine, to help KD to be buried on NE S1 site (termed EA-KD). KD and the mutant proteins were evaluated *in silico* by molecular docking to human NE, expressed in *Escherichia coli* and tested towards its NE inhibitory activity. Both mutated proteins presented enhanced NE inhibitory activity *in vitro* and RL-KD presented the best performance. We further tested RL-KD *in vivo* in an experimental model of monosodium urate (MSU)-induced acute arthritis. RL-KD showed reduced numbers of total cells and neutrophils in the mouse knee cavity when compared to KD. Nevertheless, both RL-KD and KD reduced mice hypernociception in a similar fashion. In summary, our results demonstrated that both mutated proteins showed enhanced NE inhibitory activity *in vitro*. However, RL-KD had a prominent effect in diminishing inflammatory parameters *in vivo*.

## Author summary

*Schistosoma mansoni* is one of the main agents of schistosomiasis, which is the most important human helminthic infection in terms of global morbidity and mortality. Although schistosomiasis represents a major public health problem in endemic countries, evidences show that *S*. *mansoni* downregulates inflammatory responses in many diseases. Fortunately, the control of the host inflammatory response appears not to be strictly dependent on parasite infection, but can be extended to pathogen-derived antigen, suggesting that some *S*. *mansoni* molecules are useful weapons to control inflammation. In this study we analyzed the *Schistosoma mansoni Sm*KI-1 protein and designed two different point mutations on its Kunitz Domain (KD). These mutated domains were analyzed *in silico* and expressed in *Escherichia coli* in order to evaluate their function in blocking neutrophil elastase (NE) activity and their role in inflammation using the experimental model of MSU-induced acute arthritis. Our *in vivo* data using MSU-induced acute arthritis suggest that the RL-KD mutant has a noteworthy anti-inflammatory effect compared to EA-KD mutant. Our results reinforce the growing evidence that specific molecules from parasitic helminths have immunomodulatory effects and could be an important source for developing novel therapies to control inflammatory diseases.

## Introduction

Serine proteases and their inhibitors are involved in a wide variety of cellular processes (e.g. coagulation, inflammation) and therefore, are molecules of interest when targeting therapeutic applications [1,2]. A commonly studied family of serine protease inhibitors is the one containing a Kunitz type motif. They are ubiquitous in nature [3] and target of studies in many fields (e.g. plants, human health, parasitology) due to their biological plasticity and biotechnological applicability [4]. Despite its inhibitory activity towards trypsin, chymotrypsin, elastase and other serine proteases, many studies also relate Kunitz domain containing proteins to immune system evasion by some helminth parasites [5–7]. In general, they are composed of 60–80 amino acid residues and its tertiary structure presents one α-helix and two antiparallel β-sheets stabilized by three disulfide bonds, which are connected by six highly conserved cysteine residues [6,8]. In addition, there is a positively charged residue (lysine or arginine) at the P1 position of the binding site corresponding to its enzymatic specificity [6].

The *Sm*KI-1 protein (uniprot CCD77156) is the first serine protease inhibitor functionally characterized from *Schistosoma mansoni* [9,10]. It is a 14-KDa protein with a single Kunitz-type domain (with six cysteine residues connected by 3 disulfide bonds and an arginine residue at the P1 position) and also a C-terminus domain with no similarity outside the genera [10]. To date, the *Sm*KI-1 coding sequence has been studied regarding its vaccine applicability against *Schistosoma* cercariae infection in murine model [11–13] and also regarding its anti-inflammatory and anti-thrombolytic properties [9,10]. This multi-faceted molecule has demonstrated enzymatic inhibition towards trypsin, chymotrypsin and neutrophil elastase (NE) [9,10].

We have previously reported that the recombinant *Sm*KI-1 protein inhibits neutrophil migration and reduces liver damage in a model of hepatic lesion induced by paracetamol (APAP) [10]. High anti-inflammatory potential was also observed in monosodium urate (MSU) induced gout arthritis and in carrageenan-induced pleural cavity inflammation models [10]. We also showed that the anti-inflammatory and anti-thrombolytic activities from *Sm*KI-1 are derived exclusively from its Kunitz domain (here termed KD). In the present study, we first report an optimized protocol to obtain soluble and properly folded *Sm*KI-1 KD. Additionally, in order to increase *Sm*KI-1 KD inhibitory activity, we designed two mutants (RL-KD and EA-KD), focusing on the interaction of NE pocket with KD P1 site. These proteins were analyzed for their biophysical properties and enhanced NE activity.

Our *in vitro* results reveal that both mutants, especially RL-KD, presented enhanced NE inhibitory activity when compared to the KD. Furthermore, *in vivo* data using MSU-induced acute arthritis suggest that the RL-KD mutant has a noteworthy anti-inflammatory effect worth further investigation.

## Methods

### Ethics statement

All experiments involving animals were conducted in accordance with the Brazilian Federal Law number 11.794. This law regulates the scientific use of animals in Brazil. All experiments were also in accordance with the Institutional Animal Care and Use Committees (IACUC) guidelines and the Animal Welfare Act and Regulations guidelines established by the American Veterinary Medical Association Panel on Euthanasia. All recommendations were carefully observed towards animal feeding, housing and handling. All used protocols here were previously approved by the Committee for Ethics in Animal Experimentation (CETEA/CEUA) at the Federal University of Minas Gerais (UFMG) under permit 185/2017.

### Mice and chemicals

Male TLR4^-/-^ mice aged 6–8 weeks were obtained from the Federal University of Minas Gerais (UFMG) animal facility. All reagents were purchased from Sigma-Aldrich, CO (St. Louis, MO, USA) unless otherwise specified.

### *In silico* analyses and modeling of *Sm*KI-1 Kunitz Domain mutants

Due to the nonexistence of previous experimental 3D-structures of *Sm*KI-1 protein, a hybrid approach to protein 3D-structure prediction was chosen to generate an accurate, atomistic structure of the *Sm*KI-1 Kunitz Domain (herein termed KD) as previously described by our group [10]. The model previously generated for KD [10] was used as template for two specific *in silico* site-mutations on the Kunitz Domain sequence: arginine (R18) was changed for a leucine residue (herein termed RL-KD) and the glutamic acid (E14) was changed for an alanine residue (herein named EA-KD). The model for rKD was previously submitted to preliminary molecular dynamics simulations for further analyses on the interaction of Arg^18^ and Glu^14^. MODELLER v9.22 was used for comparative homology modeling of protein structures (https://salilab.org/modeller/) for the mutations. Ten thousand structures were generated and the lowest DOPE score model was chosen. Analyzes and minimization were carried out using the software Chimera (developed by the Resource for Biocomputing, Visualization, and Informatics at the University of California, San Francisco) [14] with AMBER-ff99SB force field. This program allowed us to verify the degree of structural disruption caused by the inserted aminoacidic changes and also calculate electrostatic potential using the *APBS* tool. The tool *Surface binding analyzes* was used for electrostatic surface color rendering. The models were validated with ProCheck software (https://www.ebi.ac.uk/thornton-srv/software/PROCHECK/). The stereochemical quality of the protein structures were analyzed regarding overall residue geometry and the models were considered trustful for further analyzes.

### Molecular docking of recombinant proteins to elastase

Models built for KD and its mutants (RL-KD and EA-KD respectively) were docked to the crystal structure of neutrophil human elastase (chain A from PDB 4NZL [15]). Docking was performed in the HawkDock server (http://cadd.zju.edu.cn/hawkdock/), applying the rescoring procedure with Molecular Mechanics / Generalized Born Surface Area (MM/GBSA) and with restraints to assure docking the loop comprising residues G15-L21 from KD in the interface with Human Neutrophil Elastase (HNE) [16]. Residue 18 from KD variants were restrained to be docked within 5 Å from V219 and C198 from HNE. For each KD variant, the best scoring model for the complex with HNE was then minimized in UCSF Chimera version 1.11 [14], with addition of charges from the AMBER ff14SB force-field followed by minimization with 100 steepest descent steps (step size = 0.02 Å) and 10 conjugate gradient steps (step size = 0.02 Å). The interfaces of minimized complexes were analyzed with the software Open-Source Pymol version 1.6.x (http://www.pymol.org).

### Cloning of rKD and construction of rRL-KD and rEA-KD mutants

A construction containing the plasmid pET-32a (Novagen, Itapira, Brazil) and the coding sequence for the Kunitz domain of *Sm*KI-1 was obtained as described below. Specific primers for *SmKI-1* Kunitz domain region (S*m*KI-1 accession number: CCD77156.1) were designed according to the sequence from Gene DB (http://genedb.org) in a way signal peptide would not be amplified. For PCR isolation of the Kunitz Domain nucleotide sequence, the following set of primers were used: *KD-Forward* (5’–TTAAGAATTC**GAGAACCTGTATTTTCAAGGC**ATGCGCAAAGGTAAC–3) and *KD-Reverse* (5’–ATCTCGAGTCAGGAGCTAGTCTCGG –3’), with the restriction enzyme sites for *XhoI* and *EcoRI*, respectively, as underlined. In bold, for *KD-Forward* primer, a TEV restriction site was added. *E*. *coli XL1blue* (Agilent Technologies, Santa Clara, EUA) was transformed with the recombinant plasmid and screened on LB agar plates containing ampicillin (50 μg/mL). A selected clone was further assessed through sequencing by the Sanger Method (Myleus Biotecnologia, Belo Horizonte, Brazil) for cloning confirmation. *Sm*KI-1 Kunitz Domain mutations (RL-KD and EA-KD) were synthesized and cloned into pET-32a by GenScript (Nanjing, China).

### Expression of recombinant KD, RL-KD and EA-KD in minimum media

A clone containing the specific recombinant plasmid for each coding sequence (*KD*, *RL-KD* or *EA-KD*) was separately transformed into *E*. *coli CodonPlus* (Agilent Technologies, California, USA). Fifty milliliters of bacteria were cultured in LB media in 500 mL Erlenmeyer flasks on a rotary shaker at 180 rpm at 37°C overnight. Cells were harvested by centrifugation at 3,000 g at 4°C for 15 minutes. Cell pellet was gently washed twice with complete M9 minimum medium (42 mM Na_2_HPO_4_, 22 mM KH_2_PO_4_, 8.6 mM NaCl, 1 mM MgSO_4_, 0.1 mM CaCl, 10 μg/mL Thiamine, 0.4% w/v C_6_H_12_O_6_, 0.05% w/v NH_4_Cl, 50 μg/mL ampicillin and 52 μg/mL chloramphenicol) and then inoculated in one liter of complete M9 minimum medium on 3L Erlenmeyer flasks and cultured until OD_600nm_ of 0.9 was achieved. Gene expression was induced by adding isopropylthiogalactoside (IPTG) to the final concentration of 0.4 mM. Induction was carried out by 20 hours at 20°C at 180 rpm.

### Extraction and affinity purification of the soluble recombinant proteins

Bacterial cells were harvested by centrifugation at 3,000 g for 20 minutes and the pelleted cells were gently suspended in 50 mL of non-denaturing lysis buffer (50 mM Tris-HCl pH 8.0, 500 mM NaCl, 10 mM imidazole, 30 μg/mL Lysozyme, 1 mM Phenylmethylsulfonyl fluoride [PMSF]). Cells were then submitted to ten cycles of sonication (Branson Sonifier SLPe–Emerson Electric Co) with pulses of thirty seconds with amplitude of 30% and intervals of one minute. Solution was then centrifuged at 3,000 g for 20 minutes. Soluble recombinant proteins were recovered in the supernatant which was later used for purification by affinity chromatography on a Ni-Sepharose column (Hitrap chelating 5 mL) using an AKTA explorer chromatography system (GE Healthcare, São Paulo, Brazil) on non-denaturing conditions. PBS added 5 mM imidazole was used as running buffer and PBS added 500 mM imidazole used as elution buffer. Purified proteins were resolved using SDS-PAGE 15% (as previously described [17]) and dialyzed against PBS pH 7.0 at 4°C using a Spectra/Por 2 membrane (MWCO 6–8.000 kDa; Spectrum Medical Industries, Inc., Laguna Hills, CA). Recombinant proteins were quantified using the BCA Protein Assay Kit (Thermo Fisher Scientific, Waltham, Massachusetts, EUA) and used for further experiments.

### Thioredoxin-tag removal and RP-HPLC

Five hundred micrograms of each recombinant protein were separately incubated in 1.5 mL tubes with TEV protease at 1:100 molar rate adding 0.6 mM glutathione (GSH) and 0.4 mM glutathione oxidase (GSSG). The reaction was maintained at room temperature with overnight shaking. In order to remove precipitates. Solution was centrifuged at 17,000 g at 4° C for 30 minutes and supernatant was collected. Reverse-phase chromatography was used with a C_8_ column (5 μm; 10 x 250 mm Supelco) on a Class LC-10VP chromatography system (Shimadzu, Japan) under non-denaturing conditions for TRX removal. Supernatant was diluted in 2 mL of Milli-Q H_2_O containing 0.1% trifluoroacetic acid (TFA) and applied to a semi-preparative reverse-phase C_8_ column previously washed with acetonitrile solvent containing 0.1% TFA and equilibrated with Milli-Q H_2_O containing 0.1% TFA. The fractions were eluted after 40 and 52 minutes under a linear gradient of acetonitrile solvent containing 0.1% TFA ranging from 5 to 95% of solvent with continuous flow of 4 mL/min and collected for further purity and dosage analyzes.

### Mass spectrometry analyses and secondary structure determination

The mass / charge ratio (m/z) of the recombinant proteins from RP-HPLC were determined by MALDI-TOF/MS using linear mode on an AutoFlex III instrument (Bruker Daltonics, Billerica, USA). The samples were prepared with α-cyano-4-hydroxycinnamic matrix (1:1 molar ratio) and applied to a MALDI-TOF AnchorChip plate and crystallized at room temperature. Tris(2-carboxyethyl)phosphine (TCEP) was added to evaluate the presence of disulfide bonds [18] when needed. Samples were incubated in the presence of TCEP for thirty minutes and then applied to MALDI-TOF AnchorChip plate after crystallization with α-cyano-4-hydroxycinnamic matrix. The mass spectrometry (MS) data were obtained in MALDI-TOF by the Flex Control 3.0 software. The mass spectra were acquired with 300 laser shots at a frequency of 200 Hz and mass detection in the bands of 5,000–28,000 m/z.

Secondary structure analyses of recombinant proteins were carried out using the JASCO J-810 spectropolarimeter (Tokyo, Japan), with Peltier Jasco temperature control system—PFD425S coupled. The analyzes were performed in a quartz cuvette with 0.1 mm optical path at 20 to 50°C, with a spectral window of 190 to 260 nm. Five scans were accumulated for each curve. Data scans of buffer solutions were acquired and subtracted from protein data. Data processing and deconvolution calculations to obtain secondary structure patterns and mean residue ellipticity were performed in the Spectra Analysis, DichroWeb softwares [19] and BeStSel [20]. For DichroWeb analyzes, KD2 method was applied [21]. To obtain the thermodynamics parameters, data from the rKD protein at 218 nm (20°C to 55°C) were plotted in function of temperature and three equations were used to fit the curve and extract thermodynamics parameters.

k=exp((h/(1.987*x+273.15))*((x+273.15/TM+273.15)‐1)) (1)

y=k/(1+k) (2)

f=((u‐l)*y)+l (3)

TM represents the temperature (x) where the protein folded fraction is 50%. The variable h is the enthalpy in cal/mol; u is the mean residue ellipticity of 100% folded protein and l the mean residue ellipticity of unfolded protein. The Eq (1) calculates the folding constant. The Eq (2) the fraction folded and Eq (3) the ellipticity [22]. The first derivative of the fit curve was used to obtain the melting temperature. All spectra data were acquired and analyzed at the Proteomics Core Facility (LMProt) from Federal University of Minas Gerais (UFMG).

### 1H-NMR spectrometry

1H-NMR spectra were carried out on a Bruker AVANCE 500 MHz spectrometer operating at 500.17 MHz. The acquisition parameters to obtain a 1D spectra were: number of scans 500 at room temperature, spectral resolution 1254K using the zg protocol for hydrogen. The spectra were processed in the Bruker TopSpin 3.5 software.

### Human neutrophil elastase, trypsin and plasmin inhibitory activity assays

Inhibitory kinetics were monitored by analysis of optical density variations of NE (100 nM) (Innovative Research, Inc., Novi, MI, USA) using rKD, rRL-KD and rEA-KD as inhibitors (300 nM). Bovine serum albumin (BSA) was used as negative control. The assays were carried out with NE (100 nM) as previously described [10]. Briefly: readings were carried out up to one hour and residual enzyme activity determined at 405 nm every five minutes, using 0.2 mM N-Succinyl-Ala-Ala-Ala-p-nitroanilide substrate (Sigma-Aldrich). The initial reaction rate was determined by calculating the slope of the linear portion of the kinetic curve. The inhibitory effect was calculated and expressed as the percent reduction in the initial hydrolysis rate. Reaction rates in the absence of the inhibitors were defined as 100% for each inhibitor. The inhibitor concentration that decreased the rate of hydrolysis by 50% (IC_50_) were determined using non-linear regression with GraphPad Prism (La Jolla, CA). Results are representative of three independent experiments.

For trypsin and plasmin inhibitory activity assays, inhibitory kinetics were monitored by analysis of optical density variations of trypsin (100 nM) (Sigma-Aldrich) and plasmin (100 nM) (Sigma-Aldrich) using rKD, rRL-KD and rEA-KD as inhibitors (100 nM). Readings were carried out up to four hours and residual enzyme activity determined at 405 nm every five minutes, using 0.5 mM BApNA substrate (Sigma-Aldrich) for trypsin assays and 0.25 mM D-Val-Leu-Lys 4-nitroanilide dihydrochloride substrate (Sigma-Aldrich) for plasmin assays. Results are representative of three independent experiments.

### Gout Arthritis, nociception and inflammation parameters assessment

Joint inflammation was induced by intraarticular injection of monosodium urate (MSU) in TLR4^-/-^ mice for induced gout arthritis as a model previously described [23]. Briefly: four groups of male 8-week-old mice were used for the experiment (6–7 animals for each group: PBS control, MSU challenged, MSU challenged and rKD treated; and MSU challenged and rRL-KD treated). Each left suprapatellar ligament was treated with MSU crystal 100 μg/cavity in sterile saline for joint inflammation. The contralateral knee was treated with saline as control. All mice were sedated for the procedure. Immediately after MSU injection, groups were treated with rKD or with rRL-KD (10 mg/kg) intravenously. We used the dose of 10 mg/kg, because our group had previously tested different concentrations for rKD and decided that 10 mg/kg was the optimal dose, that's why we used this same concentration to test rRL-KD [10]. Control group was treated with saline intravenously.

For nociception assessment, mice were placed in a quiet room in acrylic cages (12x10x17 cm high) with a wire grid floor thirty minutes before the test for environmental adaptation. An electronic pressure meter was used as previously described [24,25]. Briefly: a perpendicular force was applied to the central area of the plantar surface of the hind paw and knee flexion was followed by paw withdrawal. The intensity of the pressure withstood by the mice was obtained and calculated in contrast to the non MSU treated group. The responses were representative of five readings for each animal.

Sixteen hours past joint inflammation induction, mice were euthanized. Knee synovial cavities were washed twice with 5 μL of PBS and periarticular tissues were collected. In knee synovial lavages, total leukocytes were determined by counting in a Neubauer chamber. For differential counts, leucocytes were submitted to cytospin (Cytospin 3, Shandon Inc.) and smears were stained with May-Grunwald-Giemsa, as previously described [10].

### Statistical analysis

Results from experimental groups were compared by Student’s *t*-test using the software package GraphPad Prism (La Jolla, CA). Bonferroni adjustments were included for multiple comparisons. The *p*-values obtained were considered significant when *p* < 0.05 or otherwise stated.

## Results

### *Sm*KI-1 KD mutants conception and *in silico* design

**Fig 1:**
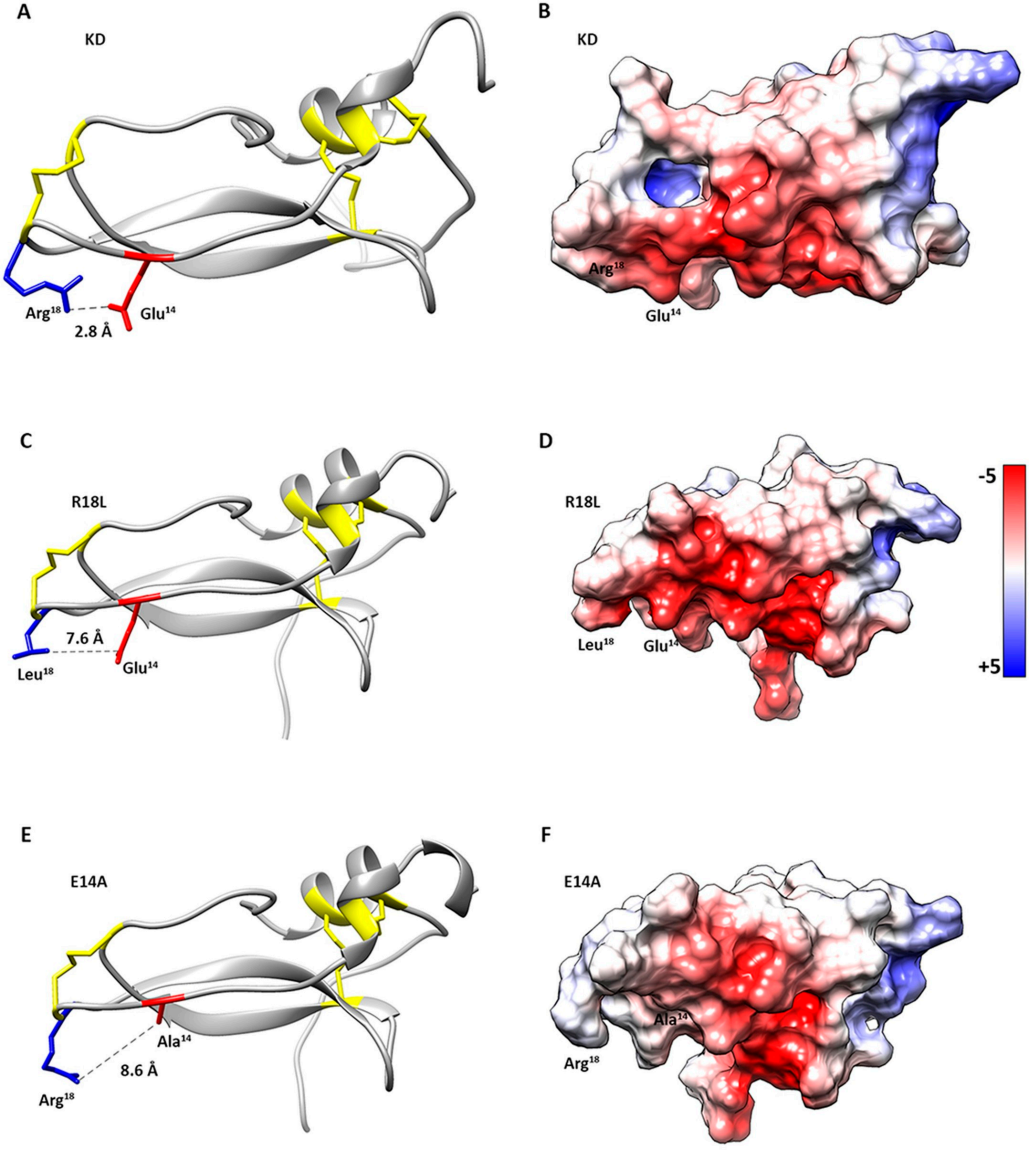
*In silico* point mutation on P1 binding site region of the *Sm*KI-1 Kunitz domain. *In silico* analyses and modeling of *Sm*KI-1 Kunitz Domain (KD) and mutants were done by a hybrid approach of protein 3D-structure prediction. Comparative homology modeling of protein structures, lowest DOPE-score model choosing and structure minimization were done by MODELLER. Analyzes were carried out using the software Chimera with AMBER-ff99SB force field. The degree of structural disruption caused by the inserted aminoacidic changes is depicted by analyzes on the interaction of R18 and E14 aminoacids, distance in angstroms and electronegativity in the protein surface (color bar represents electrostatic potentials; blue represents electropositivity and red electronegativity). Models generated for KD were used as template for two specific *in silico* site-mutations on the Kunitz Domain sequence. **A)** KD demonstrates a strong interaction between the R18 and the E14 residues, with a predicted distance of 2.8 Å. Also, both residues are surrounded by a very **B)** electronegative environment on the P1 site. **C)** The arginine on the P1 site (R18) was replaced for a leucine residue (R18L). The formerly strong interaction R18-E14 was disrupted by the mutation L18-E14 and **(D)** the P1 site seemed to become more electronegative and with a wider pocket (7.6 Å). **E)** Also, the glutamic acid (E14) was replaced for an alanine residue (E14A). The interaction R18-A14 seemed to also affect the P1 binding site **(F)** which became larger and less electronegative.

We have previously described *Sm*KI-1 Kunitz Domain 3D model through comparative homology and observed a well conserved canonical binding loops [10]. This model was used as a template for insightful point mutation to enhance KD interaction with Human Neutrophil Elastase (HNE). Our previous model revealed a strong interaction between E14 and R18 residues with a 2.8 Å distance on the P1 site (Fig 1A) and a P1 site surrounded by a very electronegative environment (total charge: -1) (Fig 1B). Also, the strong interaction observed between R18-E14 might be important to form the complex with NE allowing the R18 to enter NE S1 pocket. Our docking results indicate that the KD inhibitory loops (G15-L21 and Y38-L42) bind to the HNE active site region. Also, residues I16, R18 and L20 are docked into HNE S3, S1 and S2’ pockets, respectively. These data guided us to propose two point-mutations that were obtained by comparative homology (software Modeller v9.22).

First, the positively charged R18 at the P1 site was replaced by a leucine residue (R18L), a smaller neutral residue better suited for the binding with HNE S1 hydrophobic pocket. Secondly, we replaced the negatively charged E14 by an alanine residue (E14A), a neutral residue that might affect P1 stabilization, enhancing its flexibility and facilitating the arginine burial into the HNE S1 pocket. ProCheck assessment for the R18L mutation showed an overall adequate geometry for the model, with ~ 90% of the residues in the most favored regions and ~ 10% of the residues in the most allowed regions in the Ramachandran plot. Furthermore, E14A showed 93.2% of the residues in the most favored regions and 6.8% of the residues in the most allowed regions in the Ramachandran plot. No residues were observed in disallowed regions for either mutants and the models were found to be reliable. Chimera software was used to estimate the impact of each mutation on the recombinant KD (S1 Table).

Molecular docking for the R18L mutation demonstrated that the formerly observed strong interaction R18 and E14 was seemingly lost by the change of charge and hydrophobicity added to the system (Fig 1C). A distance of 7.6 Å was observed between those residues. This change increased the electronegativity of the P1 surrounding region (total charge: -2) when compared to KD (Fig 1D). *In silico* analysis for the E14A mutation revealed a larger distance (8.6 Å) between R18-A14 (Fig 1E) when compared to R18-E14 in the KD. The smaller alanine residue seems to contribute for a wider and less electronegative (total charge: 0) surrounding space on the binding region (Fig 1F). We propose that both mutations could enhance KD-HNE affinity, provided that inhibitors with a leucine at the P1 position are described in the literature to present enhanced NE activity (14–16).

### Molecular docking analysis reveals that point mutations on *Sm*KI-1 Kunitz domain might enhance its HNE inhibitory activity

**Fig 2:**
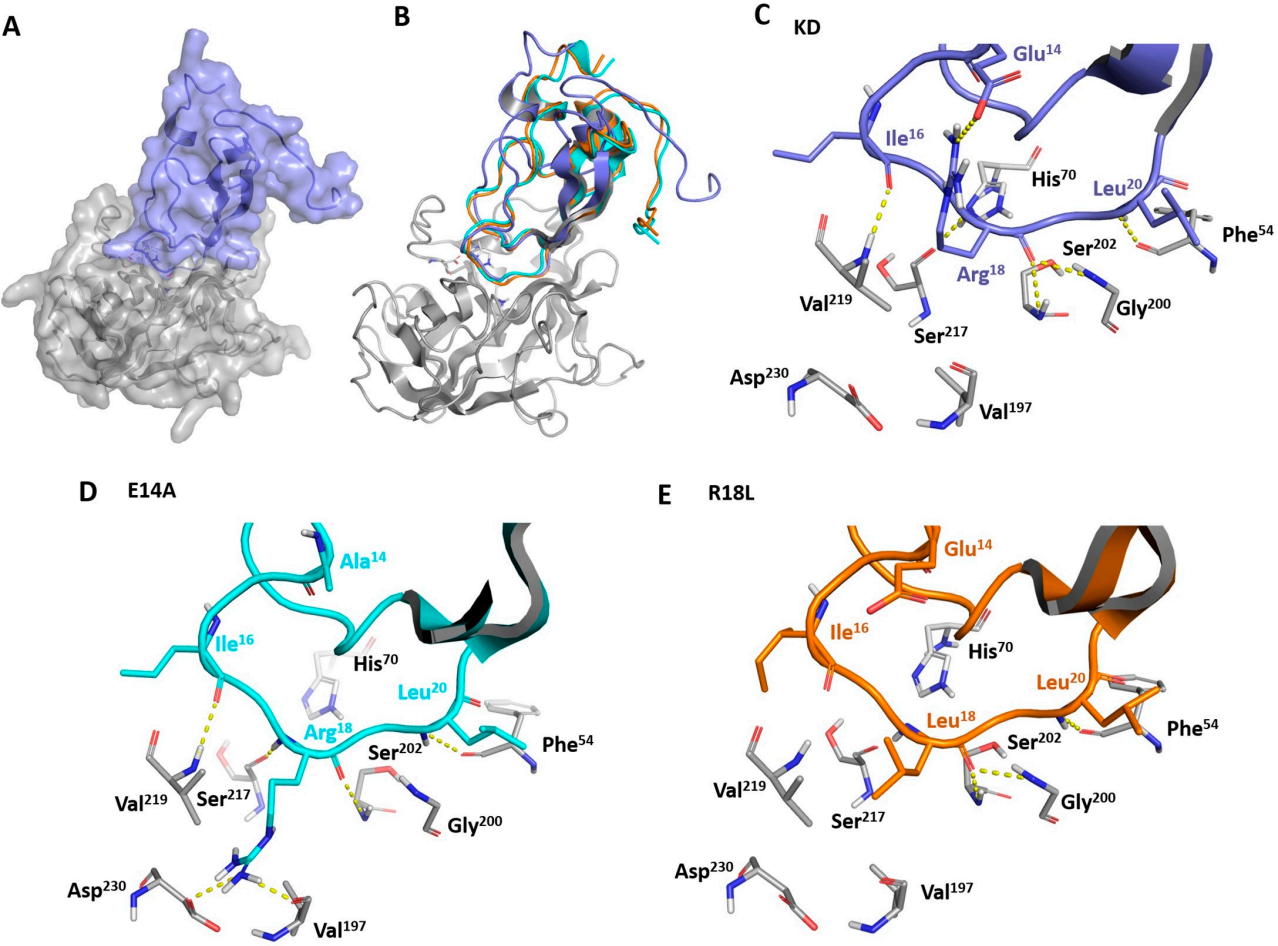
Molecular docking of *Sm*KI-1 Kunitz Domain and its variants with human neutrophil elastase. Binding mode of *Sm*KI-1 Kunitz Domain to human neutrophil elastase. **A**) Surface representation of the binding mode of KD (in purple) to human neutrophil elastase (HNE) (in gray). **B)** Superposition of the predicted binding modes for KD (purple), E14A (cyan) and R18L (orange) to HNE. Residues from elastase catalytic triad (His^70^, Asp^117^ and Ser^202^) are highlighted in sticks. Detailed analysis of the docking predicted interface between **(C)** HNE and KD, **(D)** E14A and **(E)** R18L are depicted. Residues from catalytic triad or involved in hydrogen bonds or salt bridge (yellow dashes) are represented as sticks and colored by atom, with different colors for carbon atoms of each protein: HNE (gray), KD (purple), E14A (blue) and R18L (orange). Docking predictions were performed in the HawkDock Server and figures were prepared with the software Pymol, as described in Methods.

We used the HawkDock Server in order to further investigate the *Sm*KI-1-KD-HNE complex, (Fig 2A). As previously observed for other Kunitz and WAP (wheat acidic protein) protease inhibitors [15,26,27], the predicted G15-L21 loop position is conserved for the three KD variants (KD and mutants RL-KD and EA-KD) (Fig 2B), stabilized by hydrogen bonds between the backbone atoms from HNE and the Kunitz Domain variants (S1 Table). In at least two of these three complexes, the following hydrogen bonds were predicted: KD I16-HNE V219, KD R18 (or L18) to HNE G200, S202 and S217, and KD L20 to HNE F54 (Fig 2C–2E). We also observed a conserved hydrogen bond between KD R23 and HNE N74, outside the active site region. Despite this conserved binding mode, we noticed important differences concerning the interactions involving the P1 residue. In KD (Fig 2C), R18 is predicted to form a salt bridge to E14, stabilizing this residue in a conformation in which the hydrophobic part of its side chain is directed towards the S1 pocket, while the guanidine is turned to E14. Interestingly, in the E14A mutant, the R18 side chain is predicted to bind inside S1, hydrogen bonding to V197 and forming a salt bridge with D230 inside the pocket (Fig 2D). The predicted difference in the R18 side chain position, when compared to KD might be due to the absence of E14 to stabilize the guanidine, increasing the flexibility of R18 and reducing the energetic cost of burying its side chain inside the S1 pocket. This mutation could ease the coupling of KD to HNE. In the R18L mutant (RL-KD), the L18 side chain fits into the S1 hydrophobic pocket, which contains residues F199, V219, and V197 (Fig 2E). This is in agreement with the known HNE preference for small hydrophobic residues, as reported in the MEROPS database [28], and could also be an indicative of enhanced activity favored by this mutation.

Finally, to evaluate the effect of these mutations on the energetics of the interaction with HNE, we analyzed the decomposition of the predicted binding energies by residues, reported by the HawkDock server. While for KD and for EA-KD L20 is predicted as the residue that contributes the most to the interaction, in RL-KD the L18 is predicted to have an even more favorable contribution, ranked as the most important KD residue in the interface with HNE (S2 Table).

### Samples preparation and characterization by mass spectrometry

**Fig 3:**
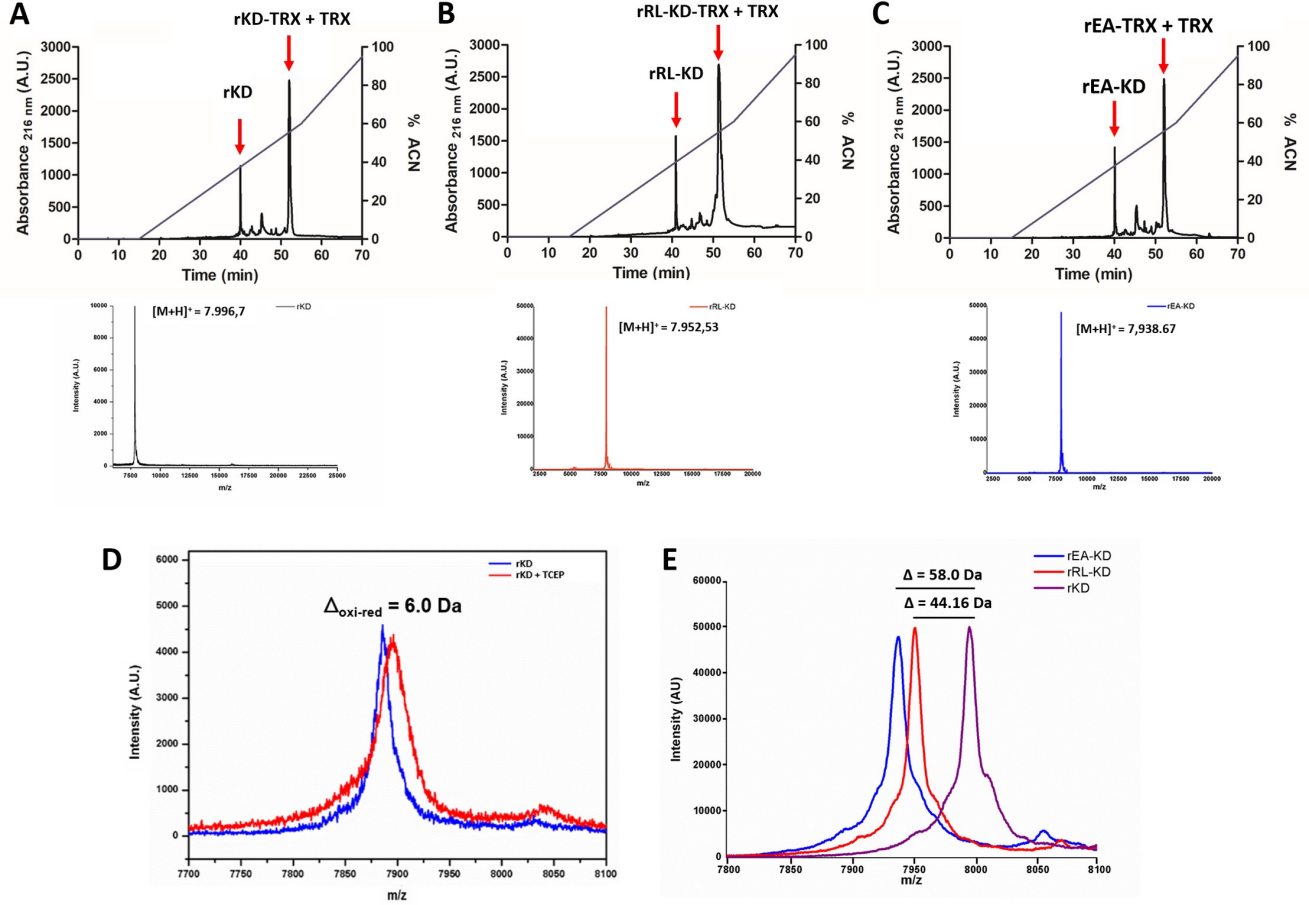
Production of soluble and functional *Sm*KI-1 Kunitz Domain and its mutants. Proteins were submitted to reverse phase high pressure liquid chromatography on a C_8_ column with acetonitrile gradient containing 0.1% TFA. Black lines represent absorbance read on 216 nm and blue lines represent percentage of acetonitrile. Red arrows point to each chromatographic fraction collected. **(A)** rKD, **(B)** rRL-KD and **(C)** rEA-KD were collected with 40 minutes (40% acetonitrile). Average molecular mass and purity of rKD was determined by MALDI-TOF/MS using linear mode on a Bruker instrument AutoFlex III. Precursor charged state [M+H]^+^ were detected at the first RP-HPLC chromatographic fraction and the observed average molecular mass was **(A)** 7,996.697 Da for rKD, **(B)** 7,952.53 Da for rRL-KD and **(C)** 7,938.67 Da for rEA-KD. **D)** Average molecular mass of TCEP reduced rKD (red lines) in comparison with oxidized rKD (blue lines) determined by MALDI-TOF/MS using linear mode was also acquired. A difference of 6 Da was observed, representing rKD is being recovered in the end of the process with its disulfide bonds connected. Also, the expected difference on observed average molecular masses from the WT Kunitz domain to the mutants were observed **(E)**.

Considering the proposed biotechnological interest implied by the R18L and E14A mutants *in silico* data, our next step was to produce soluble and functional proteins. The three coding sequences (KD and mutants RL-KD and EA-KD) were cloned into pET-32a and expressed in *Escherichia coli* as described in Methods. The obtained proteins were purified by semi-preparative reverse-phase HPLC (Fig 3A–3C) and mass analyzed by SDS-PAGE (S1 Fig) and MALDI TOF-TOF/MS for purity and average molecular mass assessment (Fig 3A–3C). The chromatographic profile of the recombinant proteins, free from the thioredoxin fusion protein (TRX), showed a distinct retention time at 40 min for rKD and rEA-KD and 41 min for rRL-KD. Non digested protein and TRX showed retention time near 50 minutes. All components were collected and further analyzed by mass spectrometry.

The analysis of the intact proteins by MALDI-TOF/MS revealed a single ion for each sample, with a molecular mass at [M+H]^+^ = 7,996.70 m/z for KD, [M+H]^+^ = 7,952.53 m/z for rRL-KD and a single ion with [M+H]^+^ = 7,938.67 m/z for rEA-KD (Fig 3C). A difference of 44.16 Da was observed when comparing rRL-KD to KD (Fig 3E). This is consistent with the expected molecular mass difference result of the replacement of an arginine residue by a leucine residue. A difference of 58.02 Da was observed when comparing rEA-KD and KD (Fig 3E). This difference is consistent with the expected molecular mass difference by the replacement of a glutamic acid residue by an alanine residue. These data suggest that the original design for the mutated proteins was achieved.

In order to assess whether the proteins were produced and recovered with their correct disulfide bonds, an experiment was conducted using Tris (2-carboxyethyl) phospine hydrochloride (TCEP) for disulfide bonds reduction and mass spectrometry (MS) (Fig 3D and 3E). In TCEP presence (red lines), disulfide bonds in the samples were reduced and a difference of 6 Da was observed when comparing to the protein in TCEP absence (blue lines) for all samples. This difference observed in the spectrum is in accordance with the amount of cysteine residues present in the protein. The disulfide bonds reduction revealed the 6 cysteine residues, which possibly acquired 6 protons gaining 6 more Da in the m/z ratio. In essence, the disulfide-rich proteins were produced in its soluble form in an *E*. *coli* system, purified with its disulfide bonds in their proper arrangement.

### Biophysical analysis revealed KD and its mutants to be soluble and well-structured proteins

**Fig 4:**
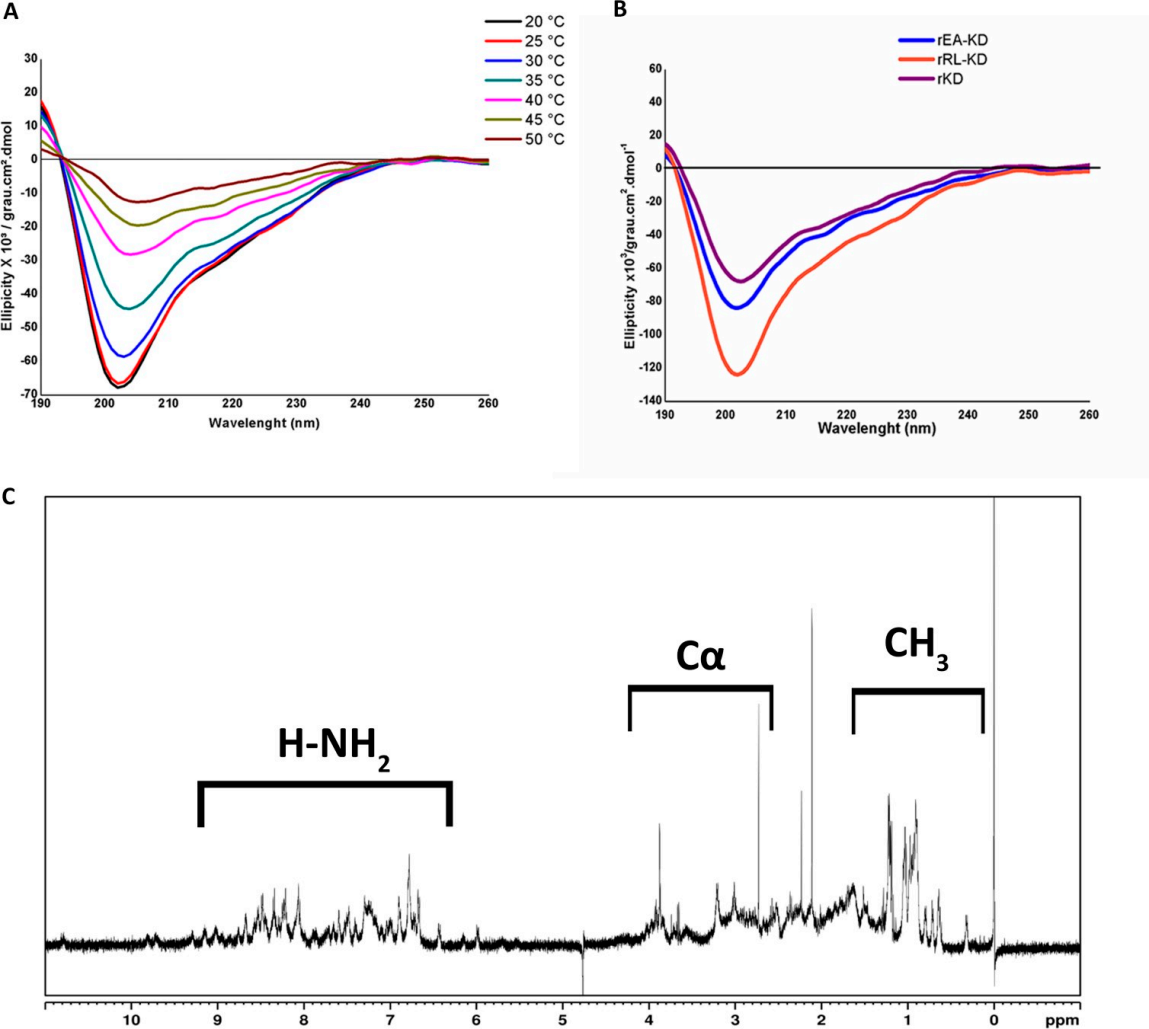
*Sm*KI-1 Kunitz Domain is recovered soluble and properly folded. rKD protein was submitted to biophysical analyzes in order to assess secondary structure evaluation. **A)** Circular dichroism analysis (far-UV spectrum) of 67 μM rKD (black lines) at 20°C shows predominant β-sheet conformation, with a classical pattern recognized on Kunitz proteins (8% α-helix, 45% β-sheet and 47% random coil content). Spectra were collected at temperatures ranging from 20–50°C showing temperature dependence on the molar ellipticity and experimental conditions are detailed in Methods. **B)** Circular dichroism analyses (far-UV spectrum) were conducted for both mutants. rKD is depicted in purple lines (8% α-helix, 45% β-sheet and 47% random coil content), rEA-KD in blue lines (8% α-helix, 42% β-sheet and 50% random coil content) and rRL-KD in orange lines (11% α-helix, 36.1% β-sheet and 52.9% random coil content). Experimental conditions are detailed in Methods. **C)** ^1^H 1D NMR spectra of rKD indicates proper folded patterns, as evidenced by the methyl side chains with resonance signals close to 0 ppm and great chemical shift dispersion of amidic hydrogens ranging from 7.5–8 ppm. The NMR spectra were acquired as described in Methods.

Finally, in order to assess the overall conformational state and stability of rKD, we performed a thermal denaturation experiment assessing changes in secondary structure by circular dichroism (CD) (Fig 4A). Far-UV CD spectrum revealed secondary structural integrity for rKD (black lines, Fig 4A) observed by predominant β-sheet conformation, with a characteristic signal for antiparallel β-sheets at 195–215 nm.

CD revealed that both rRL-KD (orange lines) and rEA-KD (blue lines) present the same predominant antiparallel beta-sheet conformation previously described [29] for the wild type Kunitz domain (Fig 4B). rEA-KD presented more similar pattern for alfa/beta content compared to rKD, as shown by deconvolution data. When comparing rKD with the mutations, molar ellipticities were slightly different at the same experimental conditions. These results reveal both mutants to maintain the Kunitz-like pattern on CD (far-UV) spectra, being recovered as properly folded proteins for further *in vitro* activity investigation. These results are in agreement with the negative minimum ellipticity observed at ~200nm, characteristic of random coil structure (47% content), and also with a slight shoulder at ~222 nm, contribution of the 8% predicted α-helix content.

To obtain thermodynamic parameters, multiple spectra were also collected for rKD on temperatures ranging from 20–55°C. The data at 218 nm in function of temperature were used to fit the Gibbs-Helmholtz equation (S2 Fig). The two-state transition of rKD from a folded to unfolded state allowed to retrieve an enthalpy of -36489.882 cal/mol. These thermodynamic data suggest a conformational change over broader folding energetics of the protein against thermal denaturation. Temperature dependence on molar ellipticity was observed, as rKD tended to unfold into random-coil and α-helix structures as higher the temperature increased, still maintaining some of its β-sheet characteristic signs.

In order to gain more detailed insights on the folding and stability of rKD we pursued the protein on NMR spectroscopy. 1D H-NMR spectra (Fig 4C) showed good dispersion in amidic hydrogen region (NH_2_) with δ between 7.5 and 8 ppm (indicated by the black square in the spectrum). We also observed methyl (CH_3_) side chains of the amino acid side chains with chemical shift (δ) close to zero in ppm (indicated by red square in the spectrum). Both signals indicate structured soluble rKD to be recovered in the end of the process.

### rRL-KD and rEA-KD presented enhanced in vitro neutrophil elastase inhibitory activity

**Fig 5:**
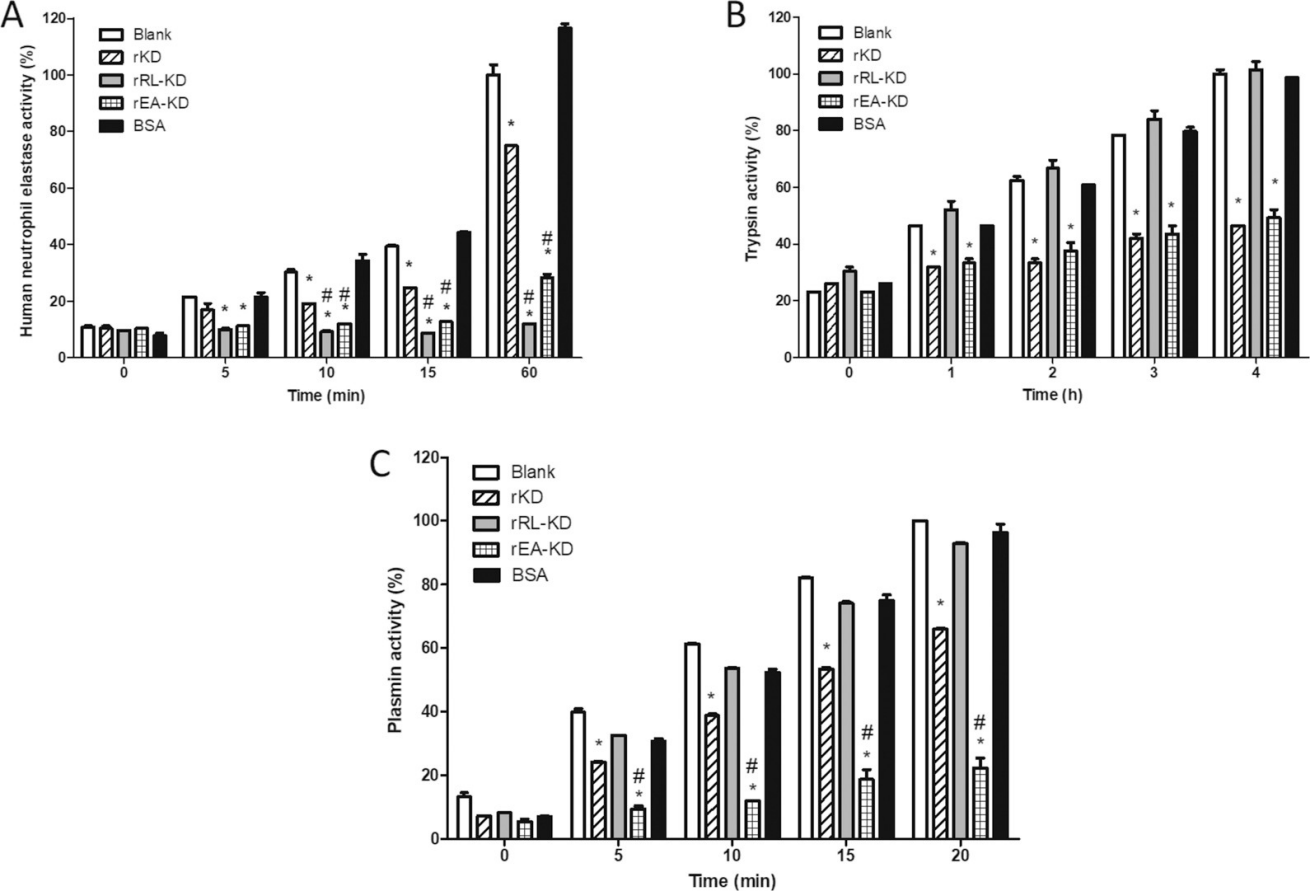
Recombinant *Sm*KI-1 Kunitz Domain mutants present enhanced neutrophilic elastase inhibitory activity *in vitro* when compared to KD. Soluble recombinant mutants RL-KD and EA-KD were tested towards their neutrophilic elastase **(A)**, trypsin **(B)** and plasmin **(C)** inhibitory activities when compared to KD. Enzymes (100 nM) were incubated at 37°C with soluble recombinant RL-KD, EA-KD and KD proteins (100nM or 300 nM) in the presence of each enzyme’s substrate. Bovine serum albumin (BSA) was used as negative control. Elastase, trypsin and plasmin inhibition activities were detected over one hour, four hours and twenty minutes of incubation with the soluble proteins, respectively. Bars indicate the mean activity for each enzyme ± standard deviation. * represents statistical difference (*p* value < 0.05) when compared to control group. # represents statistical difference (*p* value < 0.05) when compared to rKD group. rRL-KD mutant presented the best performance towards inhibition of neutrophil elastase *in vitro*, while rEA-KD presented enhanced inhibitory activity towards plasmin. These results are representative of three independent experiments.

In order to evaluate the potential of mutated proteins to inhibit NE, we performed *in vitro* enzymatic assays using the proteins (300 nM) incubated with human neutrophil elastase (100 nM) and its substrate, as described in Methods (we performed three independent replicates). The soluble proteins were assayed to its ability to inhibit NE (Fig 5A) and half-maximal inhibitory concentration (*IC*_*50*_) was calculated for the proteins (S3 Table).

As negative control, BSA was used. We observed in our previous study with r*Sm*KI-1 (full length) that its HNE inhibitory activity was present in the Kunitz domain portion (10). Our soluble rKD also demonstrated HNE inhibitory activity, as shown by the dashed bars (Fig 5A) (*IC*_*50*_: 338.8 nM). Significant differences (*p* value < 0.05 when compared to rKD) were observed for rRL-KD and for rEA-KD, as shown by gray and checkered bars, respectively. Both mutated proteins presented enhanced HNE inhibition when compared to rKD (at 10, 15 and 60 minutes of incubation in Fig 5A), as predicted by molecular docking analyzes. rRL-KD presented the best performance to inhibit HNE activity in this assay (considering mainly data at 15 and 60 minutes of incubation, Fig 5A). After a 15-minute incubation, rRL-KD showed 78% HNE inhibitory activity, while rEA-KD and rKD showed 68% and 37% HNE inhibitory activities, respectively. Considering longer periods of incubation, such 60 minutes (Fig 5A), rRL-KD showed 85% HNE inhibitory activity, while rEA-KD and rKD 72% and 25% HNE inhibitory activities, respectively. The assays demonstrated rRL-KD presented *IC*_*50*_ values of 72.3 nM while rEA-KD presented *IC*_*50*_ values of 108 nM (S3 Table).

We have also assessed the mutated proteins inhibitory activities towards other classes of serine proteases, such as trypsin (100 nM, Fig 5B) and plasmin (100 nM, Fig 5C). *In vitro* data demonstrated rRL-KD (represented by gray bars), not only showed enhanced HNE inhibitory activity (Fig 5A) but also enhanced specificity, once this molecule failed to inhibit trypsin (Fig 5B) and plasmin (Fig 5C) in the assayed conditions. On the other hand, the mutant rEA-KD (represented by checkered bars) presented no statistical difference when comparing its trypsin inhibitory activity to rKD (Fig 5B). Interestingly, rEA-KD presented enhanced plasmin inhibitory activity (Fig 5C) when compared to rKD. After a 20-minute incubation period, rEA-KD showed 78% plasmin inhibitory activity, while rKD showed 34% plasmin inhibitory activity, representing a potent molecule for further studies. The assays demonstrated rEA-KD presented *IC*_*50*_ values of 38.4 nM for plasmin while rKD presented *IC*_*50*_ values of 141.1 nM (S3 Table).

### rRL-KD *in vivo* treatment decreases inflammatory response to MSU-induced gout arthritis

**Fig 6:**
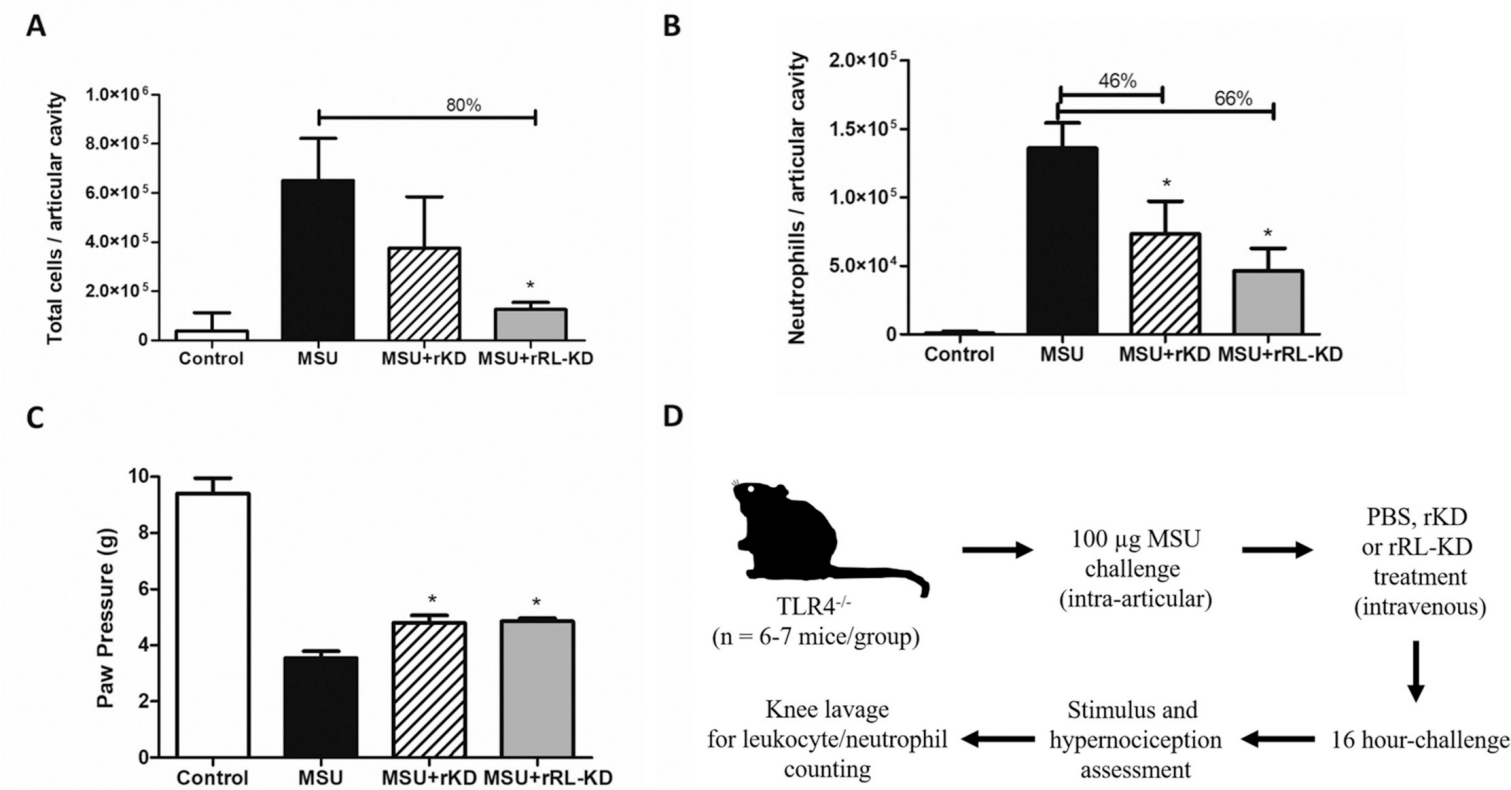
rRL-KD treatment decreased inflammation after MSU-induced acute gout arthritis. TLR4^-/-^ mice were treated with rKD or rRL-KD (10 mg/kg) or PBS vehicle i.v. 30 minutes prior to MSU injection. Animals (n = 6–7 for each group) were challenged with intra-articular knee injection of MSU crystals (100 μg/cavity). Mice were grouped as MSU control, PBS control, MSU+rKD treatment and MSU+rRL-KD treatment. Tissue inflammation was assessed by **(A)** total cells and **(B)** neutrophil recruitment in the synovial cavity. **(C)** Joint dysfunction was observed by the increase nociceptive response of mice to a mechanical stimulus by the use of an electronic paw pressure meter test made 16 hours after MSU or PBS injection. * represents statistical difference when compared to MSU group (*p* value < 0.005). **(D)** Experimental strategy is represented and further details are described in Methods. These results are representative of two independent experiments.

We have previously reported that treatment with *Sm*KI-1 reduced inflammation in animals with MSU-induced gout arthritis [10]. Since *in vitro* experimental data demonstrated rRL-KD mutated protein presented stronger NE inhibition (*IC*_*50*_: 72.3 nM), we assessed this molecule inhibitory activity in an *in vivo* experimental model of acute gout arthritis. In this model, groups of TLR4^-/-^ mice (n = 6–7 mice for each group) (we used these animals to avoid interference of any trace of bacterial LPS in the experiment) were intravenously treated with rKD or rRL-KD (10 mg/kg) or PBS (vehicle). Highly pure monosodium urate (MSU) crystals were injected into the right knee joints to induce gouty inflammation. PBS was injected in the left knee joints as control. MSU crystals induced significant accumulation of total cell numbers and neutrophils into the synovial cavity, as a measure of increased inflammatory response (Fig 6A and 6B). In contrast, rRL-KD and rKD treatments led to a reduction in the total cells counted in the articular cavity of MSU-challenged mice, but only rRL-KD treatment was observed to be statistically significant (80% reduction) (Fig 6A). This decrease was also accompanied by reduction of neutrophil infiltration in the knee cavity. rRL-KD treated animals presented a 66% reduction in neutrophil counts while rKD showed a 46% decrease (Fig 6B). However, it was not observed statistical difference when both treatments were compared. Additionally, 16 hours after MSU injection, we observed that both rKD and rRL-KD treated mice presented ameliorated mechanical hypernociception (Fig 6C). In this model, neutrophil accumulation in the articular knee cavities leads to increased hypernociception in mice [10,24]. As observed here (Fig 6C), rRL-KD or rKD treated mice presented reduced hypernociception, since they can withstand more local pressure on their paws. However, no statistical difference was observed when comparing rRL-KD to rKD protein.

## Discussion

We have shown the biotechnological potential of *Sm*KI-1 protein towards HNE inhibition *in vitro* and also towards inflammation in diverse *in vivo* experimental models [10]. In order to further investigate this molecule biotechnological potential, structural insights on its Kunitz domain were gathered by *in silico* analyzes leading us to the design of two specific mutants (R18L and E14A). *In silico* results showed the importance of the six-cysteine residues for the stabilization of the whole molecule [6] and also important sites, such as R18 and E14 at one of the inhibitory loops (DE^14^GIC^17^R^18^AL^20^LK). We considered their roles in the molecule structure especially due to R18 at the P1 site position that appears to be determinant to control its serine protease inhibitory activities [6,30]. As E14 is not as conserved as in other known inhibitors, we hypothesized its central relevance to form the complex allowing the hydrophobic site P1 to join to the active site of the NE [6,10].

Here we built homology models for both mutations (R18L and E14A) and docked them to provide insights into the interactions responsible for the binding into HNE. Docking analyzes predicted RL-KD to bind to HNE pocket the same way as L59 from EapH1 (PDB code 4NZL) [15] and L72 from SLPI (PDB 2Z7F) [27]. As a smaller neutral residue, leucine presents a tighter fit on HNE hydrophobic pocket, which contains residues Phe170, Val219, and Val197. Our *in vitro* data demonstrated rRL-KD inhibition towards NE to be significantly enhanced when comparing to rKD. This result is in agreement with the expected based on NE substrate specificity. We believe this enhanced phenotype is linked to the presence of a L18, as a hydrophobic medium-sized residue, being better accommodated by the elastase S1 pocket [28,31]. As for rEA-KD model, we observed less electronegative P1 surroundings with a wider distance between R18-A14. This happens due to the presence of a neutral residue (A14), that might fail to stabilize R18 side chain as observed by E14 in the wild-type protein. Docking of this mutant with HNE revealed that R18 flexibility led to burying of its side chain inside the HNE S1 pocket, hydrogen bonding to V197 and forming a salt bridge with D230 inside the pocket. We believe that this conformation helped enhance inhibition of NE, as we observed *in vitro* enhanced inhibitory activity toward NE when compared to rKD protein. Interestingly, analysis of the predicted energies by residues on docking data revealed that the presence of L18 at P1 was ranked as the most important residue in the interface with NE. This is in accordance with *in vitro* data, which demonstrated HNE coupling with RL-KD was much more stable than with EA-KD over time, since best performance was observed for RL-KD with one hour of incubation. Overall, aiming to develop a biotechnological product, R18L mutation revealed to be the most suitable protein in this report for further anti-inflammatory studies.

In order to perform *in vitro* experiments we used an expression system which consists on reduced rate of protein expression (due to low temperatures and minimum media**)** and consequently, minimized the formation of inclusion bodies [32,33]. This environment leads to a decrease in the speed of protein synthesis and in the number of hydrophobic interactions involved in protein aggregation [32,34]. The protocol here described was able to successfully recover the proteins in a soluble and functional fashion. 1H-NMR demonstrated a folded protein with characteristics signals for rKD while CD spectrum revealed β-sheet conformation predominance in all expressed proteins, a classical pattern for Kunitz-like proteins [29]. Interesting, rKD is very stable on the range of tested temperatures. Comparing with other proteins, rKD presents lower enthalpy change than soybean trypsin inhibitor (57000 cal/mol) from 30°C to 55°C [35] and superior to MP4 (4000 cal/mol) in the range of 15°C to 35°C [36]. We hypothesize the different parameters are directly linked to proteins specificities. A further investigation on renaturation process of rKD and the capacity of reforming the disulfide bonds will be very informative for determining protein kinetics, whereas, a comparison of TM and the mutants will be important in the understanding of its stability. As to disulfide formation, TCEP reduction coupled with mass spectrometry analysis revealed rKD to be expressed with its three disulfide bonds. These observations demonstrate this protocol produced a well-structured disulfide-rich Kunitz molecule, suitable for further structural characterization studies.

In regards to its functionality as a serine protease inhibitor, we focused on *Sm*KI-1 Kunitz domain interaction with elastase, a highly specific serine protease, which acts catalyzing the cleavage of fibrous elastin and other matrix proteins and is physiologically modulated by its inhibitors [26]. Secreted by activated neutrophils, NE is present during inflammation and, therefore, an important weapon on host defense against pathogens [23,37,38]. Neutrophils have an essential and well stablished role in the pathophysiology of inflammatory diseases, being directly correlated to disease progression and inflamed tissues. Excessive neutrophil migration to the inflammation site results in the persistent release of a number of inflammatory mediators and proteinases, including the proteolytic enzyme NE [39], contributing to progressive tissue damage. Therefore, drugs which act impairing neutrophil activity and migration (such as the *Sm*KI-1 [10]) are targets of inflammatory studies. Synthetic molecules with HNE inhibitory activity are an option for anti-inflammatory treatments, but few of them present satisfactory results [26,40]. The BF9, from *Bungarus fasciatus*, is a weakly active Kunitz-type molecule with low NE inhibitory activity [41], requiring at least 300 nM to achieve a maximum of 40% NE inhibition. Through a series of point-mutation experiments [31], authors produced several BF9-derived proteins with one of them presenting enhanced NE inhibition, requiring less than 100 nM to achieve 50% NE inhibition. The AcCI from *Apis cerana* is a chymotrypsin inhibitor with reported *IC*_*50*_ values of 38.5 nM for HNE [42], but it also presents chymotrypsin and porcine elastase inhibitions. In this work, we produced a potent and selective elastase inhibitor (rRL-KD), with *IC*_*50*_ values of 72.3 nM, an inhibitor 4.7-fold stronger than rKD. We also presented rEA-KD mutant with HNE inhibitory activity (*IC*_*50*_: 108 nM), being 3.13-fold higher than rKD’s. The full lenght *Sm*KI-1 protein has been reported by Ranasinghe and collaborators [9] with *IC*_*50*_ values of 56 nM when large enzyme/substrate molar ratios were assayed against N-Methoxysuccinyl-Ala-Ala-Pro-Val-7-amino-4-methylcoumarin substrate. Our group has also demonstrated previously this molecule activity against HNE and trypsin [10] and we observed our full length *Sm*KI-1 managed to inhibit approximately 75% HNE activity when 300 nM of the inhibitor were incubated with 100 nM of HNE. Here, 300 nM of rRL-KD managed to inhibit approximately 85% HNE activity at 60 minutes, demonstrating not only enhanced activity, but also high stability at longer periods of time.

We have also tested rRL-KD and rEA-KD inhibitory activities towards trypsin and plasmin, representing other classes of serine proteases. rEA-KD despite not presenting statistical difference to rKD inhibitory activity towards trypsin, demonstrated enhanced plasmin inhibitory activity. Plasmin is a serine protease that acts degrading fibrin and other factors from the coagulation cascade, also presenting proteinase activity for several hormones (such as adrenocorticotrophic hormone, glucagon and growth hormone) [43,44]. It is a protease with a central role in immune and inflammatory processes, since many cells express proteins on their surfaces that can act in the conversion of plasminogen to plasmin, such as monocytes, macrophages, platelets and dendritic cells [43,45,46]. Here we demonstrated rEA-KD is a strong inhibitor for plasmin (*IC*_*50*_: 38.4 nM), also presenting improved inhibitory activity for elastase, demonstrating its biotechnological potential to be explored in future works. On the other hand, rRL-KD presented itself as a potent and selective inhibitor of elastase, presenting the best performance *in vitro* towards this serine protease.

In this work we tested a model of gout arthritis, an inflammatory disease caused by the deposition of MSU crystals in the articular junctions [47]. We chose the MSU-induced acute gout experimental model to assess the recombinant RL-KD applicability as a feasible treatment for gout, comparing its efficacy to rKD. On the MSU-induced gout arthritis, a series of factors, such as cytokines, cell adhesion molecules and inflammatory cells, such as neutrophils, are recruited to the sites of the injury [23,48]. We have observed from our previous study with full length *Sm*KI-1 that MSU-induced gout arthritis mice previously treated with *Sm*KI-1 have reduced neutrophil infiltration on articular cavities followed by amelioration on hypernociception [10]. We observed here that mice treated with soluble recombinant KD or RL-KD both presented reduced hypernociception, with no statistical difference between the groups. This phenotype was followed by similar reduction on neutrophils recovered in mouse knee articular cavities. However, only rRL-KD administered animals showed significant reduction on numbers of total leukocytes recruited to the tissue. Both treatments (rRL-KD as well as rKD) presented reduction in inflammatory parameters caused by the development of gouty arthritis. When the animals were challenged with MSU and treated with RL-KD, they presented a slightly higher reduction on neutrophil and total leukocyte infiltration when compared with animals challenged with MSU and treated with KD. However, no significant difference was observed for the groups. These observations suggest the anti-inflammatory potential of rRL-KD on complex inflammatory disease models, such as gout arthritis. Further studies are needed in order to explore the mechanisms in which this molecule modulates inflammation in this model.

All recombinant proteins used in this work were obtained in *E*. *coli*, then protein concentration and LPS contamination were limiting factors in some experiments, especially those involving administration in mice. TLR4^-/-^ mice were used in order to circumvent the LPS issue when investigating the rRL-KD anti-inflammatory properties. Due to availability of animal numbers, we chose the inhibitor which presented the best performance for further *in vivo* trials. However, we need to use rRL-KD in other different inflammatory disease models besides gouty arthritis to further explore its potential.

In summary, we were able to design two mutants with enhanced *in vitro* elastase inhibitory activity, which gave us insights on function of these residues that can be used for biotechnological studies. The particular arrangement of functional amino acids conferred to this molecule new information and understanding of *Sm*KI-1 binding to HNE. Besides, it has opened up the prospect of designing new inhibitors that could target NE of variable models that are important in inflammatory processes. Despite rRL-KD mutant presented the best performance *in vitro*, further studies in inflammatory models *in vivo* are required in order to better design this molecule for anti-inflammatory therapeutic interventions.

## Supporting information

S1 Fig *Sm*KI-1 Kunitz domain wild type and mutated proteins recovery. **A)** Protein expression was induced as described in Methods and cells were harvested and lysed by mechanical and chemical disruption. Proteins were analyzed by 15% SDS-PAGE stained with Coomassie brilliant blue. The red arrow points out the rKD protein fused to thioredoxin (TRX) with approximately 26.8 kDa. kDA stands for the molecular mass ladder, NI for cell culture not induced by IPTG, L for cells disrupted by mechanical and chemical agents and S for supernatant containing soluble rKD fused to TRX (rKD-TRX). **B) S**upernatant fraction containing soluble rKD fused to TRX was submitted to affinity chromatography on a Nickel-Sepharose column and dialyzed against PBS. Ni^++^ stands for the purified Kunitz domain protein prior to rTEV protease enzymatic digestion. D stands for the Kunitz domain protein after rTEV protease enzymatic digestion. Black arrows indicate non-digested rKD fused to TRX (KD-TRX), TRX and rKD (free from TRX) proteins, with approximately 26.8 kDa, 18.95 kDa and 7.85 kDa, respectively. **C)** Mutants rRL-KD and rEA-KD protein expression were induced as described in Methods and cells were harvested and lysed by mechanical and chemical disruption. Proteins were analyzed by 15% SDS-PAGE stained with Coomassie brilliant blue. The red arrow points out the mutated proteins with the expected molecular weight. **D)** rKD, rRL-KD and rEA-KD were analyzed by 15% SDS-PAGE stained with Coomassie brilliant blue as indicated by the red arrow.
(PDF)

S2 Fig rKD ellipticity curves in function of temperature demonstrate the thermodynamics of folding. **A)** Changes in ellipticity of rKD in range of 20° to 55°C. The raw data (black line) are fit with equations for the unfolding effect (red line). **B)** The first derivative of the fitting curve showing the melting temperature.
(PDF)

S1 Table Structures validation. Ramachandran plot shows residues disposition in allowed and disallowed psi x phi correlation diagram. Proteins were evaluated using PROCHECK web server.
(PDF)

S2 Table Predicted binding energies by residues reported by the HawkDock. ^a^ ΔG values calculated in the HawkDock server, using the rescoring procedure with Molecular Mechanics/Generalized Born Solvent Area. ΔG per residues are reported only for the key residues from each protein, with the three most favorable predicted values. The * on rEA-KD column represents Arg^18^ has the 5^th^ most favorable predicted value. ^b^ Hydrogen bonds to residues from the HNE active site are highlighted in bold.
(PDF)

S3 Table Half-maximal inhibitory concentration (*IC*_*50*_) for the recombinant proteins. KD, RL-KD and EA-KD were assayed against HNE, Trypsin and Plasmin and *IC*_*50*_ were calculated with *GraphPad Prism* as described in Methods. rRL-KD fails to inhibit trypsin and plasmin and therefore is not listed on the table. 100 nM of each enzyme was used for the assays.
(PDF)
