# Correction: *S*. *mansoni Sm*KI-1 Kunitz-domain: Leucine point mutation at P1 site generates enhanced neutrophil elastase inhibitory activity

**DOI:** 10.1371/journal.pntd.0009349

**Published:** 2021-04-14

**Authors:** The PLOS Neglected Tropical Diseases Staff

There is an error in the XML that causes the last author’s name to be indexed incorrectly in PubMed. The correct initials are: Oliveira SC. The publisher apologizes for the error.
