# Supplementary figures and images for: S. mansoni SmKI-1 Kunitz-domain: Leucine point mutation at P1 site generates enhanced neutrophil elastase inhibitory activity
Source: PLoS Negl Trop Dis. 2021 Jan 19;15(1):e0009007. doi: 10.1371/journal.pntd.0009007 (PMC7846107; doi:10.1371/journal.pntd.0009007)

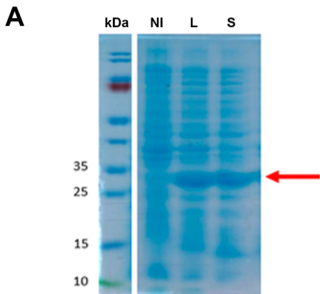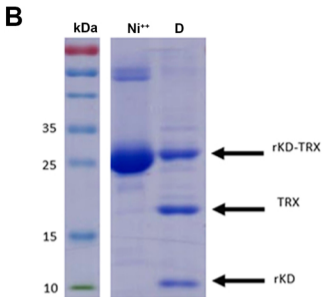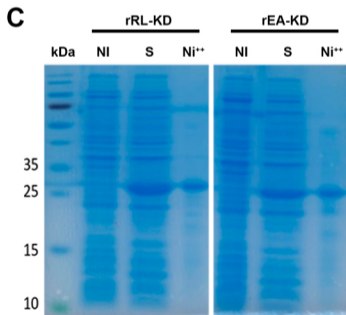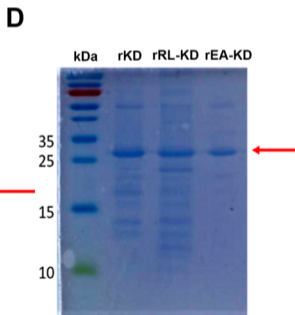

Supplement: S1 Fig — A) Protein expression was induced as described in Methods and cells were harvested and lysed by mechanical and chemical disruption. Proteins were analyzed by 15% SDS-PAGE stained with Coomassie brilliant blue. The red arrow points out the rKD protein fused to thioredoxin (TRX) with approximately 26.8 kDa. kDA stands for the molecular mass ladder, NI for cell culture not induced by IPTG, L for cells disrupted by mechanical and chemical agents and S for supernatant containing soluble rKD fused to TRX (rKD-TRX). B) Supernatant fraction containing soluble rKD fused to TRX was submitted to affinity chromatography on a Nickel-Sepharose column and dialyzed against PBS. Ni++ stands for the purified Kunitz domain protein prior to rTEV protease enzymatic digestion. D stands for the Kunitz domain protein after rTEV protease enzymatic digestion. Black arrows indicate non-digested rKD fused to TRX (KD-TRX), TRX and rKD (free from TRX) proteins, with approximately 26.8 kDa, 18.95 kDa and 7.85 kDa, respectively. C) Mutants rRL-KD and rEA-KD protein expression were induced as described in Methods and cells were harvested and lysed by mechanical and chemical disruption. Proteins were analyzed by 15% SDS-PAGE stained with Coomassie brilliant blue. The red arrow points out the mutated proteins with the expected molecular weight. D) rKD, rRL-KD and rEA-KD were analyzed by 15% SDS-PAGE stained with Coomassie brilliant blue as indicated by the red arrow. (PDF) [file pntd.0009007.s001.pdf]

**A**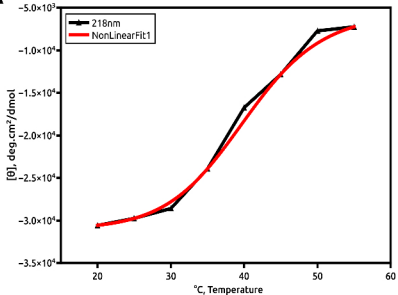**B**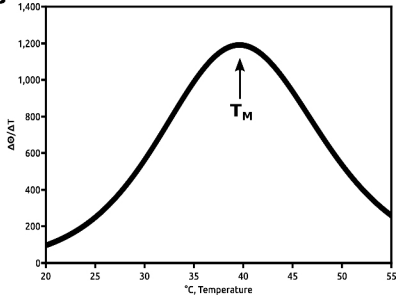

Supplement: S2 Fig — A) Changes in ellipticity of rKD in range of 20° to 55°C. The raw data (black line) are fit with equations for the unfolding effect (red line). B) The first derivative of the fitting curve showing the melting temperature. (PDF) [file pntd.0009007.s002.pdf]
