# Supplementary material for: S. mansoni SmKI-1 Kunitz-domain: Leucine point mutation at P1 site generates enhanced neutrophil elastase inhibitory activity
Source: PLoS Negl Trop Dis. 2021 Jan 19;15(1):e0009007. doi: 10.1371/journal.pntd.0009007 (PMC7846107; doi:10.1371/journal.pntd.0009007)
Supplement: S1 Table — Ramachandran plot shows residues disposition in allowed and disallowed psi x phi correlation diagram. Proteins were evaluated using PROCHECK web server. (PDF) [file pntd.0009007.s003.pdf]

S1 Table. Structures validation. Ramachandran plot shows residues disposition in allowed and disallowed psi x phi correlation diagram. Proteins were evaluated using PROCHECK web server.

	**WT-KD**	**RL-KD**	**EA-KD**
**Structural statistics**			
Ramachandran Plot PROCHECK (%)			
Most favored	79.7	88.1	88.1
Additionally allowed	18.6	10.2	11.9
Generously allowed	1.7	1.7	0
Disallowed regions	0	0	0
