# Supplementary material for: S. mansoni SmKI-1 Kunitz-domain: Leucine point mutation at P1 site generates enhanced neutrophil elastase inhibitory activity
Source: PLoS Negl Trop Dis. 2021 Jan 19;15(1):e0009007. doi: 10.1371/journal.pntd.0009007 (PMC7846107; doi:10.1371/journal.pntd.0009007)
Supplement: S2 Table — a ΔG values calculated in the HawkDock server, using the rescoring procedure with Molecular Mechanics/Generalized Born Solvent Area. ΔG per residues are reported only for the key residues from each protein, with the three most favorable predicted values. The * on rEA-KD column represents Arg18 has the 5th most favorable predicted value. b Hydrogen bonds to residues from the HNE active site are highlighted in bold. (PDF) [file pntd.0009007.s004.pdf]

S2 Table. Predicted binding energies by residues reported by the HawkDock. [a] ΔG values calculated in the HawkDock server, using the rescoring procedure with Molecular Mechanics/Generalized Born Solvent Area. ΔG per residues are reported only for the key residues from each protein, with the three most favorable predicted values. The * on rEA-KD column represents Arg^{18} has the 5th most favorable predicted value. [b] Hydrogen bonds to residues from the HNE active site are highlighted in bold.

		***Sm*KI-1 Kunitz Domain variants**		
		**rKD**	**rRL-KD**	**rEA-LD**
**ΔG (kcal/mol)[a]**		-64.48	-70.49	-71.5
ΔG per elastase residue (kcal/mol)[a]	Phe^{199}	**-8.82**	**-7.3**	**-6.74**
	Phe^{54}	-5.12	-4.31	-5.09
	Phe^{218}	-2.70	-2.74	-5.10
ΔG per *Sm*KI-1 KD residue (kcal/mol)[a]	Arg^{18} (Leu^{18})	-4.51	**-11.23**	-4.13 *
	Leu^{20}	**-7.16**	-6.33	**-7.25**
	Ile^{16}	-4.39		-4.22
	Cys^{17}		-4.20	
	Asp^{52}			-5.99
H-bons (HNE residue: KD residue: distance (Å): angle (º))[b]		**Val^{219}:Ile^{16}:3.3:166.9**		**Val^{219}:Ile^{16}:3.4:173.5**
		**Ser^{202}:Arg^{18}:3.3:147.2**	**Ser^{202}:Leu^{18}:2.9:161.6**	**Ser^{202}:Arg^{18}:3.2:131.1**
		**Gly^{200}:Arg^{18}:3.0:147.2**	**Gly^{200}:Leu^{18}:3.0:147.6**	
		**Ser^{217}:Arg^{18}:3.1:161.7**		**Ser^{217}:Arg^{18}:3.3:156.5**
		**Phe^{54}:Leu^{20}:2.8:153.7**	**Phe^{54}:Leu^{20}:2.9:155.7**	**Phe^{54}:Leu^{20}:3.2:137.5**
		Asn^{74}:Arg^{23}:2.8:110.4	Asn^{74}:Arg^{23}:2.7:153.3	Asn^{74}:Arg^{23}:2.7:160.8
			Arg^{50}:Ser^{50}:2.6:100.9	
				Arg^{50}:Asp^{52}:2.7:133.1
				Arg^{50}:Thr^{65}:2.9:112.1
				**Val^{197}:Arg^{18}:2.7:142.3**
				**Asp^{230}:Arg^{18}:3.0:147.0**
