# Supplementary material for: S. mansoni SmKI-1 Kunitz-domain: Leucine point mutation at P1 site generates enhanced neutrophil elastase inhibitory activity
Source: PLoS Negl Trop Dis. 2021 Jan 19;15(1):e0009007. doi: 10.1371/journal.pntd.0009007 (PMC7846107; doi:10.1371/journal.pntd.0009007)
Supplement: S3 Table — KD, RL-KD and EA-KD were assayed against HNE, Trypsin and Plasmin and IC50 were calculated with GraphPad Prism as described in Methods. rRL-KD fails to inhibit trypsin and plasmin and therefore is not listed on the table. 100 nM of each enzyme was used for the assays. (PDF) [file pntd.0009007.s005.pdf]

S3 Table. Half-maximal inhibitory concentration (IC_{50}) for the recombinant proteins. KD, RL-KD and EA-KD were assayed against HNE, Trypsin and Plasmin and IC_{50} were calculated with *GraphPad Prism* as described in the methods section. rRL-KD fails to inhibit trypsin and plasmin and therefore is not listed on the table. 100 nM of each enzyme was used for the assays.

**Serine protease**	**Inhibitor**	**IC_{50} (nM)**
HNE	rKD	338,8
HNE	rRL-KD	72,31
HNE	rEA-KD	108
Trypsin	rKD	101,7
Trypsin	rEA-KD	103,2
Plasmin	rKD	141,1
Plasmin	rEA-KD	38,41
